# Managing vertical dimensions in patients with Amelogenesis Imperfecta: A case report

**DOI:** 10.1002/ccr3.6135

**Published:** 2022-08-22

**Authors:** Warren Farao, Imaan A. Roomaney

**Affiliations:** ^1^ Department of Conservative Dentistry, Faculty of Dentistry University of Western Cape Cape Town South Africa; ^2^ Department of Craniofacial Biology, Faculty of Dentistry University of Western Cape Cape Town South Africa

**Keywords:** Amelogenesis imperfecta, attrition, dentistry, occlusal vertical dimensions, prosthodontics

## Abstract

Amelogenesis imperfecta (AI) is a heterogeneous group of conditions characterized by inherited developmental defects of enamel. Patients with AI often have progressive and severe loss of occlusal vertical dimensions (OVD), resulting in challenging dental rehabilitation. In this case report, we present the management of a 24‐year‐old male patient who previously underwent orthodontics, direct and indirect restorations, and continued to have progressive tooth wear. His vertical dimensions were restored in two phases, firstly with provisional restorations at the improved OVD, followed by a combination of monolithic zirconia and lithium disilicate full‐coverage crowns. A removable acrylic appliance was then constructed to protect his teeth. This report emphasizes the importance of preserving and protecting the OVD from an early age to prevent more costly and complicated management in future.

## Abstract 2

Progressive loss of OVD is a common problem in patients with AI. This results in challenges in restoring aesthetics and function. To mitigate this, protection and restoration of the OVD should be prioritized in the dental management of patients with AI.

## 1 BACKGROUND

Amelogenesis imperfecta (AI) is a heterogeneous group of conditions characterized by inherited developmental defects of enamel.^1^ The prevalence of AI varies widely among populations, with reports ranging from 1 in 700 to 1 in 14,000 people affected.^1^, ^2^, ^3^ AI significantly impacts the Oral health‐related quality of life (OHRQoL) of those affected due to the aesthetic and functional problems caused by the dental phenotype.^4^, ^5^ Enamel is abnormally thin, soft, fragile, pitted, or discolored, and patients are prone to early tooth loss.^1^ This causes patients to be self‐conscious, have eating difficulties, and suffer from chronic pain.^6^

In 1991, it was discovered that the *AMELX* gene was involved in the development of AI.^7^ Since then, at least 18 genes have been implicated in non‐syndromic AI, and many more have been associated with AI as part of a syndrome.^1^, ^3^ More genes involved with the development of AI are likely to be discovered due to the increased accessibility of next‐generation sequencing (NGS). These genes play various roles in amelogenesis, calcium homeostasis, and other functions associated with normal enamel development. Thus, pathogenic variants in these genes have a wide range of phenotypic consequences which can broadly be classified as (1) hypomaturation AI, (2) hypomineralized AI, (3) hypocalcified AI, and (4) mixed AI.^1^, ^2^, ^6^ Additionally, teeth with AI often undergo rapid post‐eruptive changes,^3^ which makes identifying the type of AI challenging.^1^ Irrespective of the type of AI, the patient usually requires complex, lifelong, and costly dental management from a multidisciplinary dental team.^8^, ^9^

Occlusal vertical dimensions (OVD) is the distance between two selected anatomic points when the mandibular teeth are occluding with the maxillary teeth.^10^ In patients with AI, reduced OVD results from a combination of factors, including reduced tooth size, agenesis of teeth, tooth loss, malocclusion, parafunction, and qualitative and quantitative defects in the enamel.^11^ The loss of OVD is a frequent challenge in patients with AI and becomes progressively more difficult to manage as patients age.^10^ In this case report, we present a young adult patient that has undergone extensive dental treatment and presents with progressive loss of OVD. The treatment options and management of the patient are discussed.

## 2 CLINICAL REPORT

A 24‐year‐old male patient presented to the Prosthodontic Department at the University of Western Cape with a main complaint of stains and the progressive breakdown of his teeth. He experienced these problems for 3 years before seeking assistance. The patient requested an improvement in his appearance and a long‐term solution to his deteriorating dentition.

The patient's dental history revealed a diagnosis of AI. He had undergone fixed orthodontic treatment as a teenager. His four impacted wisdom teeth were surgically removed 7 years prior. Thereafter, ten all‐ceramic crowns were placed. He regularly attended the general dentist for preventative care. Written consent was obtained from the patient for his management and the publication of this case report.

**FIGURE 1:**
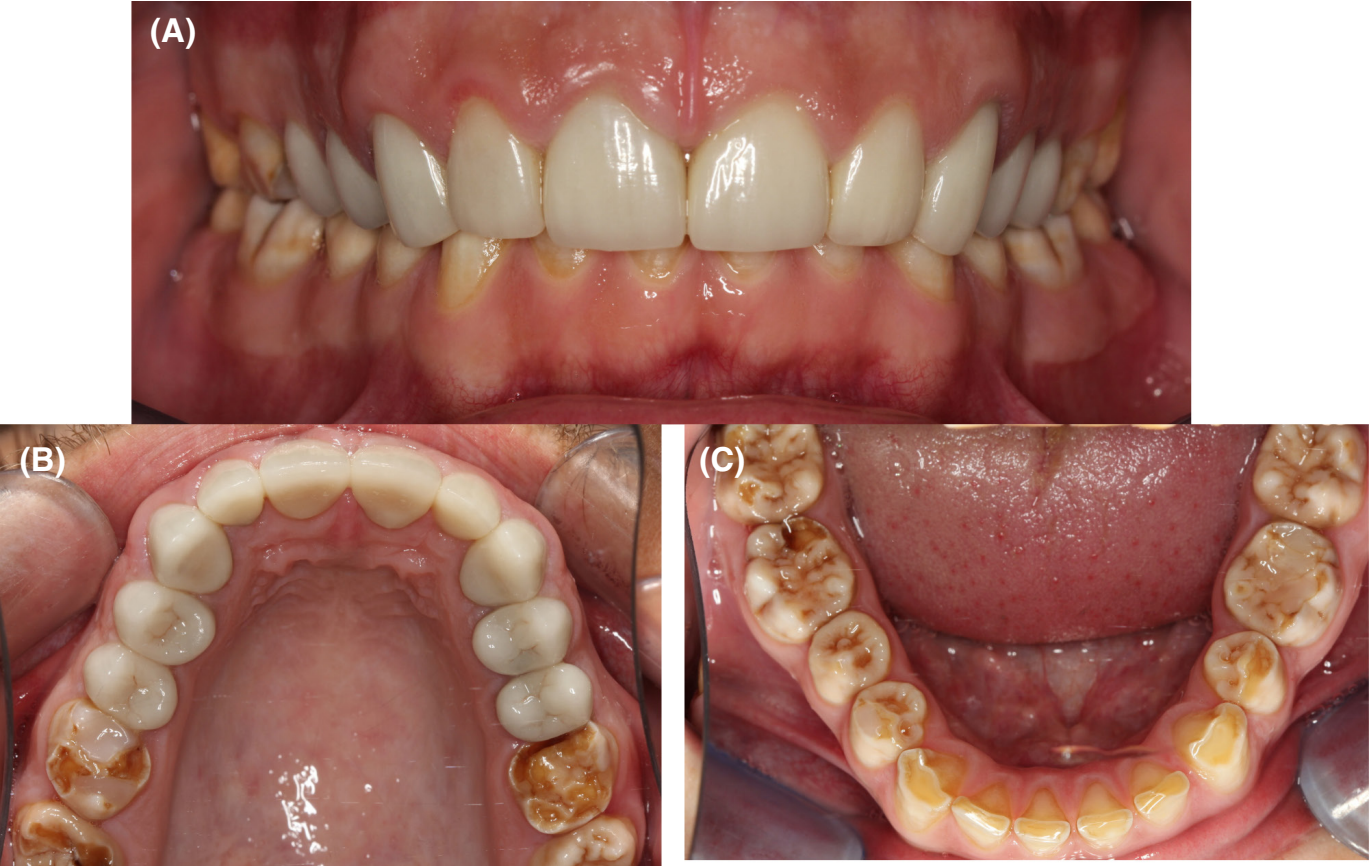
(A–C): Intraoral images of the patient with AI. (A) The dentition in occlusion. Note the deep bite and lack of lower incisor teeth showing. (B) The maxillary arch showing ceramic full‐coverage crowns 15–25 and defective restorations and wear on the molars. (C) The mandibular arch with AI affecting all teeth and moderate wear

Extra‐oral examination revealed good facial symmetry with an equal distribution of facial thirds. He had a straight profile with sufficient lip support in the maxilla and mandible. No temporomandibular joint (TMJ) abnormalities were detected. He presented with a prominent, high smile line, excessive maxillary teeth, and minimal to no mandibular anterior teeth showing during occlusion. Individual tooth examination showed all‐ceramic crowns on teeth 15–25, and generalized AI (Figure 1). The mandibular incisors and canines had visible attrition (Figure 1C) and brown‐stained AI affected enamel on all occlusal surfaces. Defective resin restorations were present on teeth 16 and 36. Generally, his oral hygiene was good. OVD was measured by an experienced prosthodontist using a Willis gauge and confirmed with extra‐oral markers on the nose and chin, taken at rest and occlusion. Freeway space (FS) was calculated to be 5 mm. Although this is only slightly out of the normal range,^11^ the loss of OVD was visually apparent.

**FIGURE 2:**
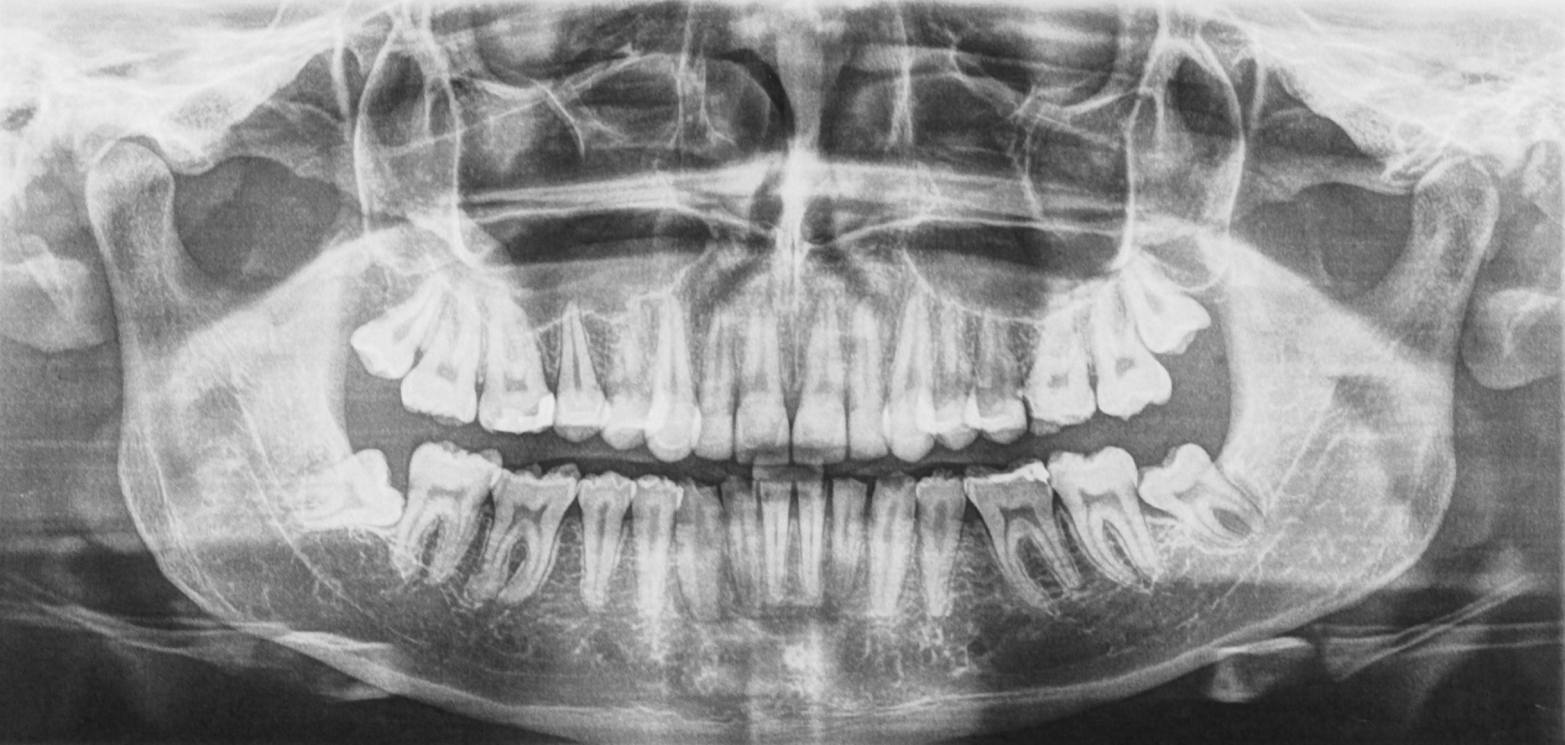
Preoperative Panorex showing caries on the 16, 26, 35, 45, 46, and 47, and congenitally missing 35. The 18, 28, 38, and 48 were impacted

Pre‐treatment radiographs (Figure 2) revealed no gross bony pathologies. Pneumatization of the maxillary sinuses was noted on both left and right sides. Caries were detected on 16, 26, 35, 45, 46, and the 47. The 35 was congenitally missing, and the wisdom teeth (18, 28, 38, and 48) were impacted. The cephalometric analysis showed a Steiner Skeletal Class I relationship (ANB of 4°) and Wits Skeletal Class II relationship (3 mm).

A diagnosis of hypomaturation AI was made based on the presence of moderate to severe wear of the posterior teeth without crowns, mottled agar‐brown tooth discolouration, and visible differences between the radiographic enamel and dentine. Additional diagnoses included a borderline Skeletal Class II relationship (Steiner = ANB 4°, Wits = 3 mm), dental caries, attrition, loss of OVD, and a congenitally missing 35.

Treatment goals were aimed at (1) improving and maintaining the patients' oral health status, (2) restoring defective restorations and caries, (3) restoring the aesthetic profile and function, (4) providing an optimal occlusal scheme at an improved vertical height, and (5) preventing further deterioration of the vertical dimensions and the dentition.

### 2.1 Diagnostic and provisional phase

**FIGURE 3:**
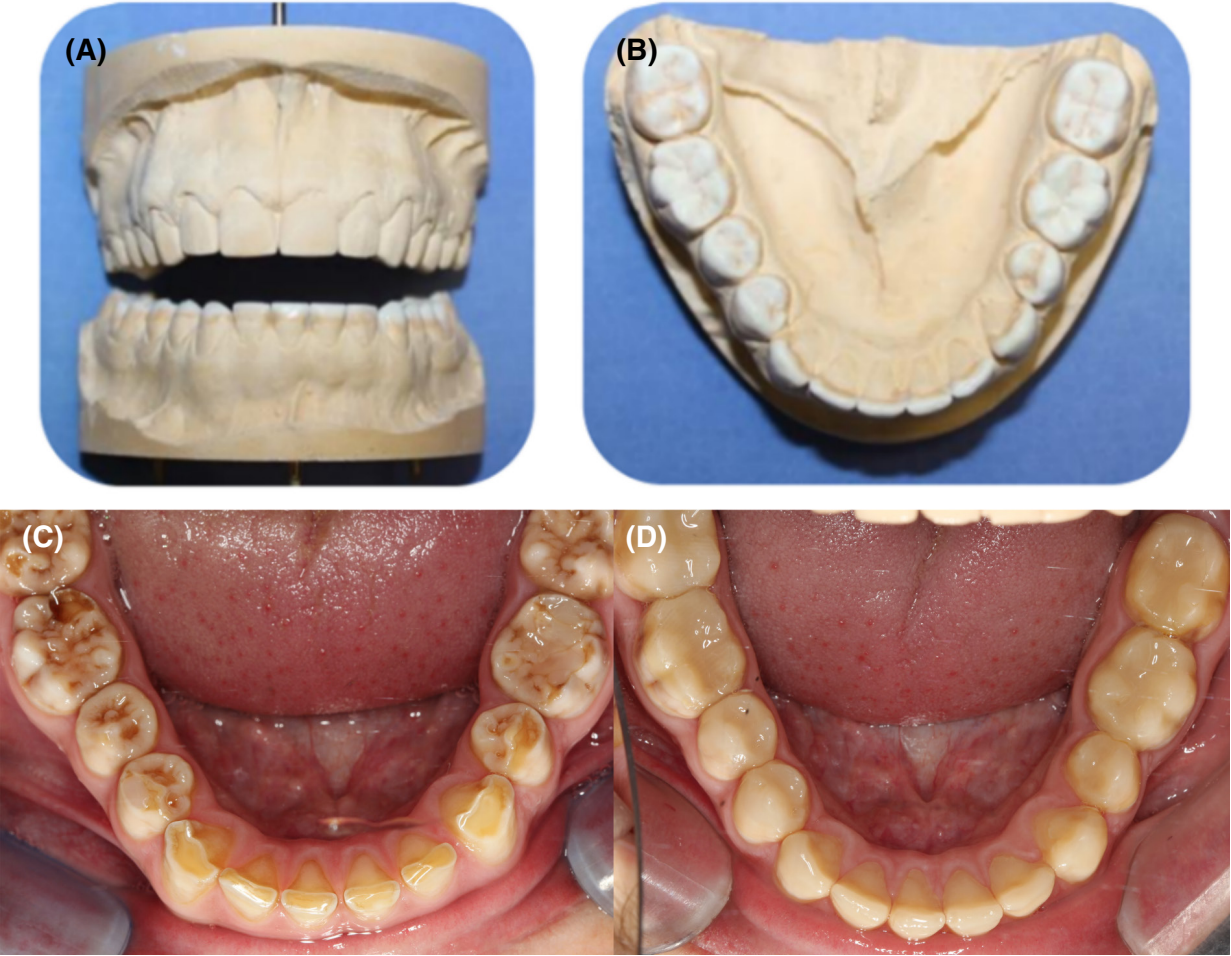
(A–D): (A & B) Diagnostic wax‐up (At improved OVD) (C) Preoperative mandibular dentition showing wear (D) Provisional resin build‐up phase

The dental laboratory prepared study casts for a diagnostic wax‐up (Figure 3). Instructions were given to the laboratory to increase the OVD by 2 mm anteriorly. The diagnostic wax‐up was used to create a template for the construction of a provisional mock‐up with composite resin. This allowed for the evaluation of aesthetics and phonetics. These restorations were also used as provisional restorations.

### 2.2 Restorative phase

The definitive treatment plan included all‐ceramic crowns made from monolithic zirconia on the 16, 26, and 46. All‐ceramic crowns on the 33 and 43 were to be constructed from lithium disilicate. We opted for veneers on the 42, 41, 31, and 32. A protective occlusal appliance and six‐monthly maintenance visits were planned for the patient. The patient rejected the inclusion of periodontal surgery and restorative work on the maxilla to improve the “gummy smile,” which would have included the replacement of existing all‐ceramic crowns (15–25) after surgical crown lengthening.

**FIGURE 4:**
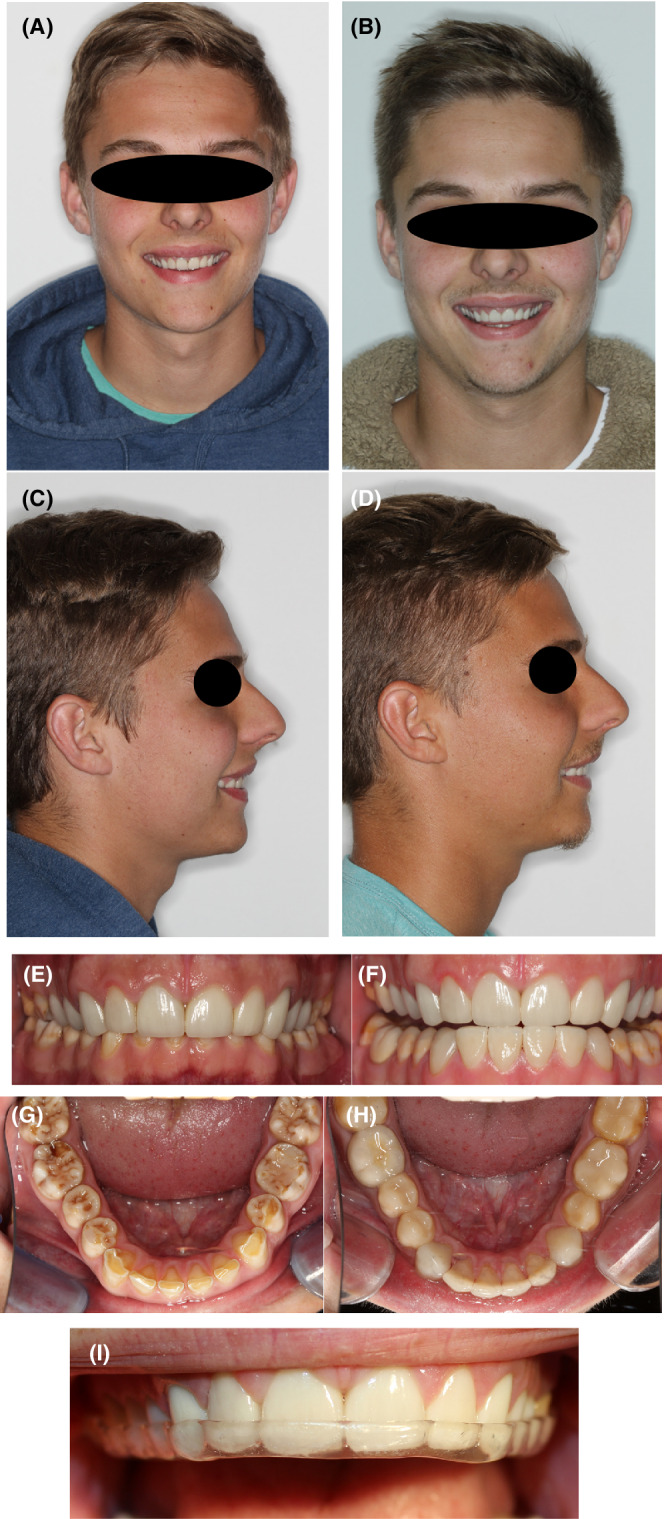
Pre‐ and post‐operative comparative photographs showing the improvement in aesthetics and vertical dimensions. (A & B) Pre‐treatment frontal and profile images; (C & D)Post‐treatment frontal and profile images showing an improvement in aesthetics; (E & F) Pre‐ and post‐treatment frontal views of the dentition; (G & H) Pre‐ and post‐operative occlusal views of the mandibular teeth; (I) Acrylic occlusal removeable maxillary appliance to protect the dentition

Final impressions were taken using soft polyether impression material (Impregum™ Penta 2™ 3M™) in conjunction with the single retraction cord technique. The completed restorations (monolithic zirconia) were checked for accuracy of fit, the occlusion was evaluated, and the aesthetics and phonetics were assessed. After conditioning the restorations with hydrofluoric acid and silane treatment, the crowns were bonded with a self‐adhesive cement (Rely – X Unicem, 3M™). An occlusal analysis was conducted with the patient performing lateral excursive movements to identify non‐working contacts. A hard, acrylic protective occlusal appliance was delivered to protect the restorations. Pre‐ and post‐operative comparative images are presented in Figure 4.

## 3 DISCUSSION

In this case report, we present the prosthodontic management of a young adult with AI who has experienced progressive OVD loss. Although the patient has had comprehensive dental management from an early age, there was insufficient protection of the occlusion, suggesting that the OVD was not thoroughly considered during earlier management. Hence, we emphasize the importance of protecting the OVD as soon as patients with AI are identified to mitigate more complex dental treatment in future.

Dentists often adopt a “wait‐and‐see” approach when treatment planning in young patients with AI. This is due to previous management guidelines which encouraged delaying definitive prosthodontic therapy in children and adolescents until adulthood was reached.^12^, ^13^ Given the phenotypic diversity of the condition, it is somewhat understandable that the clinician would want to be conservative in their management approach. However, without adequate preservation of the dentition in patients with AI, there is likely to be excessive tooth wear, the development of a deep vertical bite and a reduction in mesiodistal tooth widths, resulting in more complex and costly dental management.^8^

In recent years, there has been a paradigm shift to support the use of more long‐term restorative modalities such as crowns in younger patients with AI to prevent the complications caused by tooth loss and reduced OVD.^14^, ^15^ Studies by Lungren et al. found the longevity of direct composite dental restorations was significantly lower in patients with AI, with 35.1% of the AI group requiring replacement of fillings during the observation period compared to 23.7% of the control group.^16^ As expected, porcelain crowns had a significantly longer survival time than composite resin materials used in the AI group. A recent study evaluating the cost feasibility also found that the placement of crowns in children and adolescents leads to decreased long‐term costs, equating to fewer restoration replacements and dental visits.^17^ Additionally, the OHQoL was significantly higher in those with crowns instead of composite restorations.^17^

Both direct and indirect treatment options are regularly suggested for the restorative rehabilitation of patients with AI. Indirect treatment options include minimally invasive methods such as veneers and partial coverage restorations, or classical methods such as crowns and fixed dental prostheses (FDPs). Depending on the indication, composite, ceramic materials, and alloys may be used to fabricate the indirect restorations.^18^ Unfortunately, no guidelines currently exist for the dental management of patients with AI in low‐resource settings, such as ours. The cost of crowns often exceeds patients' affordability. Instead of long‐term treatment planning, patients are often treated as problems arise, complicating long‐term management. As with the presented case, composite resin restorations are often used as long‐term restorations and merely replaced as required. These restorations have a high rate of replacement and frequent marginal leakage.^16^ Aesthetics is often inferior to crowns.^4^ Thus, more innovative management solutions need to be explored in these circumstances. An example of innovative management would be prefabricated composite veneers which can be done in a single visit and require minimal removal of tooth structure.^19^ Fortunately, the presented case had access to prosthodontic management. Thus, the failing posterior restorations could be replaced with crowns which have a high clinical success rate in teeth affected by AI.^15^ We chose to use monolithic zirconia to restore his posterior teeth due to the strength requirements of the molars and premolars. The canines and incisors were restored with a lithium disilicate which has superior aesthetic properties. The butt‐joint design, with the addition of a palatal chamfer, was selected as the most appropriate veneer preparation due to the worn tooth structure.^20^

Indications for treating tooth wear typically include compromised aesthetics, sensitivity, pain and discomfort, occlusal instability, difficulties chewing, and wear compromising tooth vitality.^21^ A critical consideration when restoring worn dentitions is determining the amount of the OVD lost due to tooth wear. In cases of physiological wear, it is acknowledged that the OVD can be maintained due to the teeth's over eruption and the alveolar bone growth compensation. Alternatively, pathological tooth wear is faster than the compensatory mechanism, which accompanies a loss of OVD. As a result, side effects include aesthetic problems, decreased masticatory function, and TMJ disease due to loss of anterior and lateral guidance.^22^

The existing OVD can be assessed extra‐orally and intraorally. It is critical to verify the loss of OVD before the reconstruction of an increased OVD. The different techniques that can be employed to evaluate OVD loss are phonetics, the evaluation of interocclusal distance and the evaluation of soft tissue contours.^23^ In this case, the wear on the mandibular teeth was due to the softer nature of the enamel and the opposing ceramic restorations of the anterior maxillary teeth, indicating the need for an occlusal appliance. Occlusal appliances have several uses, including but not limited to the following^24^:

1. To temporarily provide an occlusal condition that allows the TMJs to assume the most orthopedically stable joint position.
2. To introduce an optimum occlusal condition that reorganizes the neuromuscular reflex activity, which in turn reduces abnormal muscle activity while encouraging more normal muscle function.
3. To protect the teeth and supportive structures from abnormal forces that may create breakdown and/or tooth wear—this was the reason for us using a protective occlusal appliance in this case.

It should be noted that the patient presented still requires the remaining posterior teeth with resin build‐ups to be replaced with zirconia crowns. Treatment is costly, especially in low‐resource settings, and efforts must be made to lessen the costs of therapy for the affected patients.

## 4 CONCLUSION

Patients with AI require complex, costly, lifelong dental management. Protection of the OVD is often overlooked when managing patients. This report emphasizes the importance of holistically managing the dentition and prioritizing protecting the occlusion from an early age in patients with AI.

## AUTHOR CONTRIBUTIONS

WF was responsible for the conceptualization, patient management, and drafting of the case report. IAR assisted in the conceptualization, drafting, review and editing of the case report. Both authors are jointly responsible for the work presented.

## FUNDING INFORMATION

IAR is supported through funding by the South African Medical Research Council through its Division of Research Capacity Development under the SAMRC Institutional Clinician Researcher Development Programme from funding received from the South African National Treasury. The content hereof is the sole responsibility of the authors and does not necessarily represent the official views of the SAMRC or the funders.

## CONFLICT OF INTEREST

None to declare.

## CONSENT

Written consent has been obtained from the patient for management and publication of this case report.

## Data Availability

The data that support the findings of this study are available on request from the corresponding author. The data are not publicly available due to privacy or ethical restrictions.
